# Association of visceral adiposity index with hypertension (NHANES 2003–2018)

**DOI:** 10.3389/fcvm.2024.1341229

**Published:** 2024-05-09

**Authors:** Haoran Zhou, Tianshu Li, Jie Li, Dongdong Zheng, Jie Yang, Xin Zhuang

**Affiliations:** ^1^ Shandong University of Traditional Chinese Medicine, Jinan, China; ^2^ Department of Cardiology, The Affiliated Hospital of Shandong University of Traditional Chinese Medicine, Jinan, China

**Keywords:** VAI, hypertension, NHANES, cross-sectional study, association

## Abstract

**Objectives:**

This study focused on the association between visceral adiposity index (VAI) and the prevalence of hypertension in a nationally representative population of American adults.

**Methods:**

The study obtained data from the National Health and Nutrition Examination Survey (NHANES) database from 2003–2018 for a large-scale study. This study incorporated participants ≥18 years of age. Multivariate logistic regression modelling and smoothed curve fitting were applied to investigate the existence of a correlation between VAI and hypertension prevalence. Subgroups were analyzed to confirm the stationarity of the association between VAI and hypertension prevalence. In addition, an interaction test was conducted in this study.

**Results:**

In completely adapted sequential models, the risk of hypertension prevalence in the overall population increased 0.17-fold with each 1-unit increase in VAI [odds ratio (OR) = 1.17; 95% confidence interval (CI) 1.12–1.22]. In the wholly adapted categorical model, there was a 0.95-fold increased risk of hypertension in the population of VAI quartile 4 (Q4) vs. VAI quartile 1 (Q1) (OR = 1.95; 95% CI 1.62–2.35). These results indicate that VAI was strongly related to the occurrence of hypertension, and smoothed curve-fitting analysis showed nonlinearity. Adjustment for covariates revealed no apparent interactions in the subgroup analyses, and results were stable across subgroups.

**Conclusion:**

This cross-sectional study suggests a nonlinear and positive correlation between elevated VAI and the adult risk of developing hypertension in U.S. adults.

## 1 Introduction

Hypertension is a common chronic disease with high prevalence and is one of the most prevalent risk elements for cardiovascular disease (CVD) (1, 2). According to epidemiologic statistics, there are approximately 1 billion hypertensive patients worldwide, and it is projected that the population of hypertensive patients will increase to 1.292 billion by 2025 (3, 4). In the United States, it was found that the prevalence of hypertension in adults is about 32%–46% (5). Among those aged 60 years and above, the percentage of women suffering from hypertension is about 75% (6). The global incidence of hypertension has been gradually increasing in recent years, and the age of hypertension has been decreasing. Hypertension is the disease that causes the most significant number of deaths in the world and causes severe damage to the heart, brain, and kidneys. At the same time, hypertension itself and its complications cause suffering to patients and a substantial financial burden to families. The prevention, treatment, and management of hypertension has become an urgent issue in the field of public health and medical care.

Risk considerations for hypertension include high sodium and low potassium intake in the diet, smoking and use of alcohol in lifestyle behaviours, psychological stress, sleep disorders, obesity and overweight (7). Hypertension due to obesity and overweight has become a hot issue widely studied by scientists, and its prevalence has increased significantly in the United States and worldwide. Among the many complications of obesity, hypertension is the most common and significant complication, accounting for approximately 70% of the obese population (8). Measures of obesity include body mass index (BMI), waist circumference (WC), and hip circumference (9, 10). However, there are limitations in clinical application, such as BMI is incapable of evaluating body fat percentage, and WC is incapable of distinguishing between visceral adipose tissue and abdominal subcutaneous adipose tissue (11). The visceral adiposity index (VAI) has been proposed to address this issue. VAI is an indicator of visceral adiposity related to cardiometabolic status and has been shown to correlate with visceral adipose tissue area and volume independently of subcutaneous adipose tissue (12). Hence, VAI, which has been introduced as a surrogate indicator of adipose tissue function, could more directly predict the progression and risk of cardiovascular disease (13, 14). Numerous studies have found VAI to be positively associated with insulin resistance (IR), kidney stones, heart failure, and depression (15–18). In addition, epidemiologic studies exploring the relationship between VAI and the risk of hypertension have shown that VAI is positively linked to the risk of hypertension in the Chinese population (19) and that there is a nonlinear positive association between VAI and cardiovascular disease (20). In contrast, other studies have found no correlation with gender (21). There are no extensive cross-sectional studies on the association between VAI and the risk of hypertension in the United States.

Therefore, the purpose of the current investigation is to detect the potential link between VAI and the prevalence of hypertension in U.S. adults through the data collected by NHANES between 2003 and 2018 to provide a scientific basis for the prevention, diagnosis, treatment, and of hypertension as well as to provide strong evidence for the results of previous studies.

## 2 Materials and methods

### 2.1 Study population

**Figure 1:**
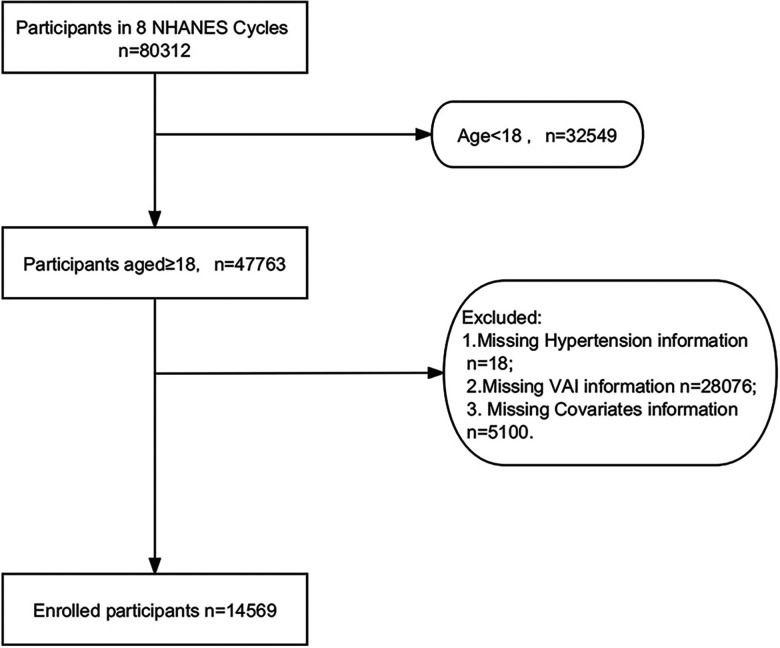
The flowchart of participants.

NHANES is an extensive cross-sectional survey designed to assess the health and nutritional status of adults and children in the United States. Our data are all obtainable from the NHANES website (https://www.cdc.gov/nchs/nhanes/index.htm), all participants signed informed consent forms, and the project was endorsed by the National Center for Health Statistics (NCHS) Ethics Review Board. We analyzed data from the last eight cycles (2003–2018). A total of 80,312 subjects were enrolled in 8 consecutive NHANES investigative cycles between 2003 and 2018, including 14,569 participants. The reasons for excluding other participants were as follows: 1. 32,549 aged <18 years; 2.18 missing information on hypertension; 3. 28,076 missing information on the VAI index; and 4. 5,100 missing information on covariates (Figure 1).

### 2.2 Definition of VAI

NHAENS blood sample collection and measurements are performed according to standardized protocols from the Centers for Disease Control and Prevention (CDC). A dedicated staff is available to organize the information, specimen testing, and analysis.VAI assesses visceral fat using anthropometric data (BMI, WC) and biochemical indicators (TG, HDL-C). The formula was calculated as follows: VAI = [WC/(39.68 + 1.88 × BMI)] × (TG/1.03) × (1.31/HDL) for men, and VAI = [WC/(36.58 + 1.89 × BMI)] × (TG/0.81) × (1.52/HDL) for women. BMI = body weight (kg)/height (m^2^), WC units are cm, TG and HDL units are mmol/L.

### 2.3 Definition of hypertension

NHANES organized experienced investigators to record participants' blood pressure based on the American Heart Association guidelines (22). The specific blood pressure measurements are all available on the official NHANES website. In the resting state for at least 5 min, we performed three consecutive blood pressure measurements (each at least 1 min apart) to obtain their average values. Hypertension was defined as (i) self-reported hypertension, (ii) current use of antihypertensive medication, and (iii) mean systolic blood pressure (SBP) ≥ 140 mmHg and/or mean diastolic blood pressure (DBP) ≥ 90 mmHg.

### 2.4 Covariates

Based on the methodology of previous studies, we selected variables such as demographic data, socioeconomic status, lifestyle, physical examination, laboratory tests, and personal history as potential confounders to estimate the relation between VAI and hypertension. Covariates comprised age, gender, ethnicity, education, marital status, household poverty-to-income ratio (PIR), smoking, alcohol consumption, BMI, WC, total cholesterol (TC), high-density lipoproteins (HDL), low-density lipoproteins (LDL), triglycerides (TG), fasting blood glucose (FPG), hemoglobin A1c (HbA1c), and estimated glomerular filtration rate (eGFR), diabetes mellitus(DM), coronary heart disease(CHD), antihypertensive medications, and antiHyperlipidemic medications. Participants were categorized into five racial groups: Mexican American, non-Hispanic white, non-Hispanic black, other Hispanic, and other races. Age (18–34, 35–54, 55–74, ≥75), sex (male/female), level of education (less than 9th grade, 9th–11th grade, high school graduation, some college graduation, college and above), marital status (married, widowed, divorced, separated, unmarried, living with a partner), PIR (<1.3, 1.3–3.5, ≥3.5), and cigarettes (previous, current, non-smoking), alcohol consumption (previous, light/moderate, heavy, non-drinking). Diabetes mellitus (yes/no), coronary heart disease (yes/no), antihypertensive drugs (yes/no), antihyperlipidemic drugs (yes/no).

### 2.5 Statistical analysis

All data in this study were statistically analyzed by applying R language (version 4.3.1). Continuous variables were represented as mean ± standard deviation, and categorical variables were represented as frequency (percentage). Differences between categorical variables were analyzed using the chi-square test, while continuous variables meeting normal distribution were tested using the weighted Student's t-test; otherwise, the Mann-Whitney u-test was used. We quadruple-categorized the VAI. Multivariate logistic regression models were applied to investigate the relevance of VAI to hypertension after adjusting for potential confounders. The degree of correlation was represented as OR and 95% CI. Three models were developed: Model 1 was not adjusted for confounders; age, gender, and ethnicity were adjusted in Model 2, and Model 3 further adjusted for level of education, marital status, PIR, tobacco use, alcohol use, eGFR, DM, CHD, antihypertensive drugs, and antihyperlipidemic drugs. Smoothed curve fitting was then applied to examine the existence of a nonlinear relationship between VAI and hypertension. Ultimately, subgroup analyses of the confounders listed in the baseline table (age, sex, race, education, marriage, eGFR, smoking, alcohol use, DM, and CHD) were performed using hierarchical logistic regression modelling for the presence of interactions. The threshold for a statistically significant difference was set at *P* < 0.05.

## 3 Results

### 3.1 Baseline characteristics

The characteristics of all participants are shown in Table 1. A total of 14,569 (Figure 1) participants ≥18 years of age were included in this research. Hypertension was present in 37.66% of the participants, and the weighted mean age was 47.49 ± 0.25 years. Among them, 49.50% were males, and 50.50% were females. Patients at higher risk for hypertension tended to be female, ≥50 years of age, non-Hispanic white, married, less educated, lower household PIR, higher BMI and WC, higher lipid and glucose levels, and higher alcohol and tobacco use. In addition, patients at higher risk for hypertension were more likely to have diabetes and coronary heart disease. They were divided into four groups (Q1-Q4) according to the VAI quartiles. The differences in age, race, level of education, marital status, PIR, smoking, alcohol consumption, BMI, WC, FPG, HbA1c, TC, TG, LDL, HDL, eGFR, DM, CHD, antihypertensive medication, and antihyperlipidemic medication among the four groups of patients were statistically significant (*P* < 0.05).

**Table 1:** The characteristics of participants.

Variables	Total	Q1	Q2	Q3	Q4	*P*-value
	(*n* = 14,569)	(*n* = 3,645)	(*n* = 3,639)	(*n* = 3,643)	(*n* = 3,642)	
Age (years)	47.49 ± 0.25	44.44 ± 0.48	46.73 ± 0.34	48.67 ± 0.35	50.25 ± 0.34	<0.0001
Gender, *n* (%)						0.07
Female	7,256 (50.50)	1,701 (48.23)	1,795 (50.80)	1,863 (52.04)	1,897 (51.01)	
Male	7,313 (49.50)	1,944 (51.77)	1,844 (49.20)	1,780 (47.96)	1,745 (48.99)	
Race, *n* (%)						<0.0001
Mexican American	2,296 (7.90)	385 (6.04)	508 (7.18)	657 (8.85)	746 (9.60)	
Non-Hispanic Black	2,884 (9.96)	1,105 (15.08)	805 (10.89)	604 (8.62)	370 (5.07)	
Non-Hispanic White	6,761 (70.72)	1,516 (67.66)	1,667 (70.66)	1,709 (70.57)	1,869 (74.08)	
Other Race	1,382 (6.60)	401 (7.02)	336 (6.07)	343 (7.08)	302 (6.23)	
Others Hispanic	1,246 (4.82)	238 (4.20)	323 (5.20)	330 (4.89)	355 (5.03)	
Education, *n*(%)						<0.0001
9–11th grade	2,050 (10.40)	451 (8.46)	482 (9.83)	525 (10.77)	592 (12.60)	
College graduate or above	3,451 (30.01)	1,106 (38.08)	944 (31.41)	771 (26.53)	630 (23.73)	
High school graduate	3,378 (23.52)	736 (19.76)	850 (23.69)	885 (24.19)	907 (26.56)	
Less than 9th grade	1,425 (4.99)	218 (3.19)	319 (4.51)	391 (5.63)	497 (6.68)	
Some College	4,265 (31.09)	1,134 (30.52)	1,044 (30.55)	1,071 (32.88)	1,016 (30.43)	
Marital status, *n*(%)						<0.0001
Divorced	1,596 (10.53)	362 (9.47)	392 (10.30)	408 (10.68)	434 (11.71)	
Living with partner	1,163 (7.97)	312 (8.70)	303 (8.41)	269 (7.73)	279 (7.00)	
Married	7,673 (56.81)	1,752 (54.00)	1,896 (55.60)	2,007 (57.98)	2018 (59.78)	
Never married	2,526 (17.04)	886 (22.01)	668 (18.37)	546 (15.75)	426 (11.85)	
Separated	466 (2.22)	113 (1.83)	106 (2.29)	124 (2.34)	123 (2.43)	
Widowed	1,145 (5.43)	220 (3.99)	274 (5.03)	289 (5.51)	362 (7.24)	
PIR						<0.0001
<1.30	4,309 (19.82)	942 (17.15)	1,001 (18.76)	1,099 (20.33)	1,267 (23.13)	
1.30–3.50	5,619 (36.18)	1,387 (34.43)	1,406 (35.74)	1,448 (37.89)	1,378 (36.73)	
>=3.50	4,641 (44.00)	1,316 (48.42)	1,232 (45.50)	1,096 (41.77)	997 (40.13)	
eGFR, ml/min/1.73 m^2^	94.61 ± 0.34	98.42 ± 0.56	95.15 ± 0.45	93.15 ± 0.46	91.56 ± 0.52	<0.0001
BMI, kg/m^2^	28.87 ± 0.09	25.67 ± 0.12	28.09 ± 0.16	30.04 ± 0.15	31.80 ± 0.14	<0.0001
WC, cm	99.04 ± 0.23	89.99 ± 0.30	96.76 ± 0.40	102.23 ± 0.32	107.51 ± 0.36	<0.0001
FPG, mmol/L	5.84 ± 0.02	5.50 ± 0.02	5.63 ± 0.02	5.90 ± 0.03	6.35 ± 0.05	<0.0001
HbA1c, %	5.59 ± 0.01	5.38 ± 0.01	5.48 ± 0.01	5.63 ± 0.02	5.85 ± 0.03	<0.0001
TC, mmol/L	5.02 ± 0.01	4.78 ± 0.02	4.94 ± 0.02	5.07 ± 0.02	5.31 ± 0.02	<0.0001
TG, mmol/L	1.35 ± 0.01	0.67 ± 0.00	1.01 ± 0.01	1.40 ± 0.01	2.36 ± 0.02	<0.0001
HDL, mmol/L	1.41 ± 0.01	1.78 ± 0.01	1.48 ± 0.01	1.29 ± 0.01	1.09 ± 0.01	<0.0001
LDL, mmol/L	2.97 ± 0.01	2.66 ± 0.02	2.98 ± 0.02	3.12 ± 0.02	3.13 ± 0.02	<0.0001
VAI	1.83 ± 0.02	0.60 ± 0.00	1.13 ± 0.00	1.84 ± 0.01	3.80 ± 0.03	<0.0001
Smoking status, *n*(%)						<0.0001
Former	3,713 (25.75)	828 (23.56)	909 (24.88)	949 (26.19)	1,027 (28.46)	
Never	7,842 (53.73)	2,170 (59.72)	2,012 (55.61)	1,919 (51.98)	1,741 (47.39)	
Now	3,014 (20.52)	647 (16.72)	718 (19.51)	775 (21.83)	874 (24.15)	
Alcohol use, *n*(%)						<0.0001
Former	2,465 (13.85)	442 (9.10)	568 (13.08)	662 (14.56)	793 (18.82)	
Heavy	2,907 (20.82)	705 (20.13)	759 (22.41)	751 (21.55)	692 (19.18)	
Mild/Moderate	7,259 (54.96)	2,072 (61.97)	1,843 (53.61)	1,724 (53.32)	1,620 (50.70)	
Never	1,938 (10.38)	426 (8.79)	469 (10.90)	506 (10.57)	537 (11.29)	
CHD, *n*(%)						<0.0001
No	13,958 (96.41)	3,538 (97.38)	3,508 (97.14)	3,485 (96.42)	3,427 (94.64)	
Yes	611 (3.59)	107 (2.62)	131 (2.86)	158 (3.58)	215 (5.36)	
DM, *n*(%)						<0.0001
No	11,707 (85.17)	3,254 (93.07)	3,093 (89.67)	2,838 (83.36)	2,522 (74.25)	
Yes	2,862 (14.83)	391 (6.93)	546 (10.33)	805 (16.64)	1,120 (25.75)	
AntiHyperlipidemic, *n*(%)						<0.0001
No	11,665 (81.75)	3,142 (88.03)	2,988 (84.65)	2,818 (79.09)	2,717 (74.97)	
Yes	2,904 (18.25)	503 (11.97)	651 (15.35)	825 (20.91)	925 (25.03)	
AntiHypertensive, *n*(%)						<0.0001
No	9,928 (72.28)	2,825 (82.72)	2,583 (75.77)	2,351 (68.95)	2,169 (61.29)	
Yes	4,641 (27.72)	820 (17.28)	1,056 (24.23)	1,292 (31.05)	1,473 (38.71)	
Hypertension, *n*(%)						<0.0001
No	8,393(62.34)	2,488(74.57)	2,179(65.45)	1,961(58.54)	1,765(50.36)	
Yes	6,176(37.66)	1,157(25.43)	1,460(34.55)	1,682(41.46)	1,877(49.64)	

PIR, poverty-to-income ratio; eGFR, estimated glomerular filtration rate; BMI, body mass index; WC, waist circumference; FPG, fasting plasma glucose; HbA1c, hemoglobin A1c; TC, total cholesterol; TG, triglyceride; HDL, high-density lipoprotein; LDL-c, low-density lipoprotein-cholesterol; CHD, coronary heart disease; DM, diabetes mellitus.

### 3.2 Multifactorial logistic regression analysis

Table 2 shows the relationship between VAI and hypertension incidence. Three models were constructed: model 1, model 2, and model 3. VAI was used as a continuous variable, and in model 1 (Unadjusted model), the prevalence of hypertension increased by 29% with each unit increase in VAI, with an effect value OR and 95% CI of 1.29 (1.24, 1.33), respectively. Model 2 adjusted for age, gender, and ethnicity and had an effect value OR and 95% CI of 1.26 (1.21, 1.30), respectively. Model 3 continued to adjust for education, marriage, PIR, smoking, alcohol consumption, eGFR, DM, CHD, antihypertensive medication, and antihyperlipidemic medication based on model 2, with an effect value OR and 95% CI of 1.17 (1.12, 1.22), respectively. VAI was changed from a continuous variable to a categorical variable and categorized into four levels based on quartiles of VAI. Using Q1 as a control, the prevalence of VAI and hypertension exhibited a monotonically increasing trend in all models (*P* < 0.0001). In conclusion, VAI is positively linked to the development of hypertension.

**Table 2:** Relationship between VAI and hypertension in different models.

Character	Model 1		Model 2		Model 3	
	OR (95% CI)	P	OR (95% CI)	P	OR (95% CI)	P
Per 1 unit increase %	1.29 (1.24,1.33)	<0.0001	1.26 (1.21,1.30)	<0.0001	1.17 (1.12, 1.22)	<0.0001
VAI group						
Q1 (0 < VAI ≤ 0.870)	1(ref)		1(ref)		1(ref)	
Q2 (0.870 < VAI ≤ 1.420)	1.55 (1.34,1.78)	<0.0001	1.48 (1.27,1.73)	<0.0001	1.39 (1.15, 1.68)	<0.001
Q3 (1.420 < VAI ≤ 2.371)	2.08 (1.80,2.39)	<0.0001	1.90 (1.64,2.20)	<0.0001	1.60 (1.33, 1.92)	<0.0001
Q4 (2.371 < VAI)	2.89 (2.50,3.34)	<0.0001	2.57 (2.21,2.99)	<0.0001	1.95 (1.62, 2.35)	<0.0001
*p* for trend	<0.0001		<0.0001		<0.0001	

Model 1: non-adjusted.

Model 2: adjutesd age, gender and race.

Model 3: adjutesd age, gender, race, education, marital, PIR, smoke, alcohol, eGFR, DM, CHD, antiHyperlipidemic and antiHypertensive.

PIR, poverty-to-income ratio; eGFR, estimated glomerular filtration rate; DM, diabetes mellitus; CHD, coronary heart disease.

### 3.3 Curve fitting analysis

**Figure 2:**
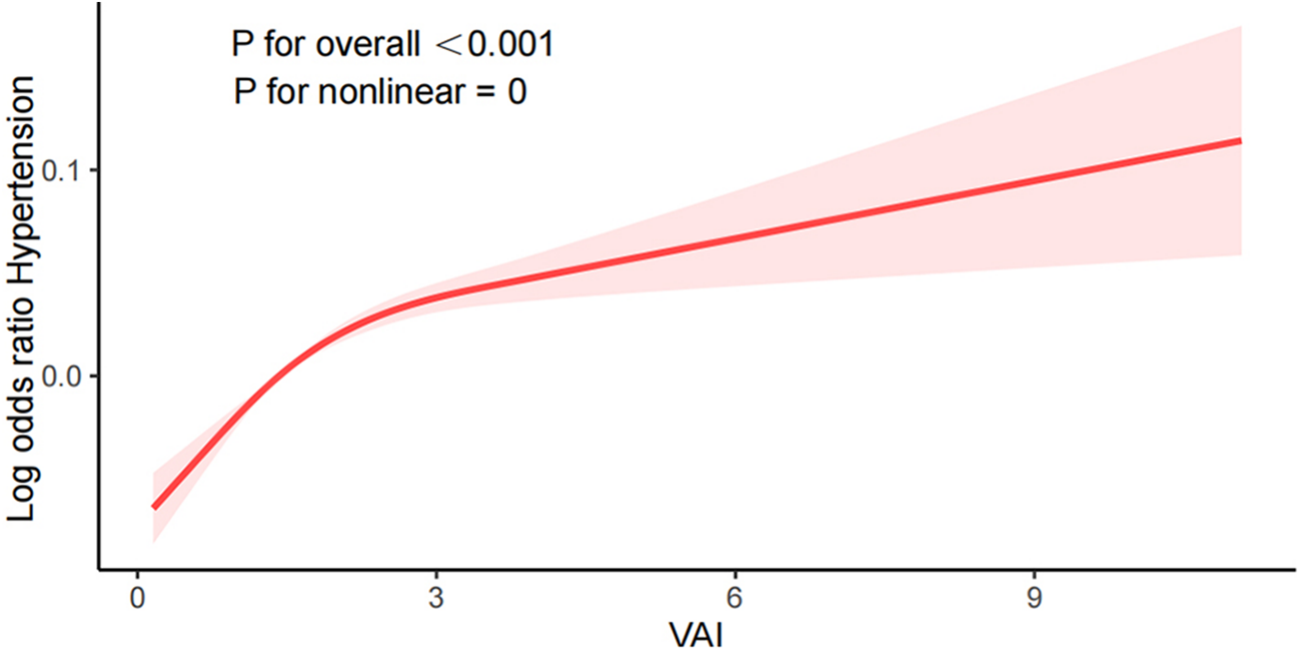
Association between visceral adiposity index and hypertension.

Smooth curve fitting plots, like those shown in Figure 2, visualize the connection between VAI and the prevalence of hypertension. The nonlinear association between VAI and hypertension(*P* for nonlinear <0.001) was observed after adjusting for confounders such as age, gender, ethnicity, level of education, marital status, PIR, smoking, alcohol consumption, eGFR, DM, CHD, antihypertensive drugs, and antihyperlipidemic drugs. The risk of hypertension showed a nonlinear positive increasing trend with increasing VAI, and the trend was steeper with larger VAI.

### 3.4 Subgroup analysis

**Figure 3:**
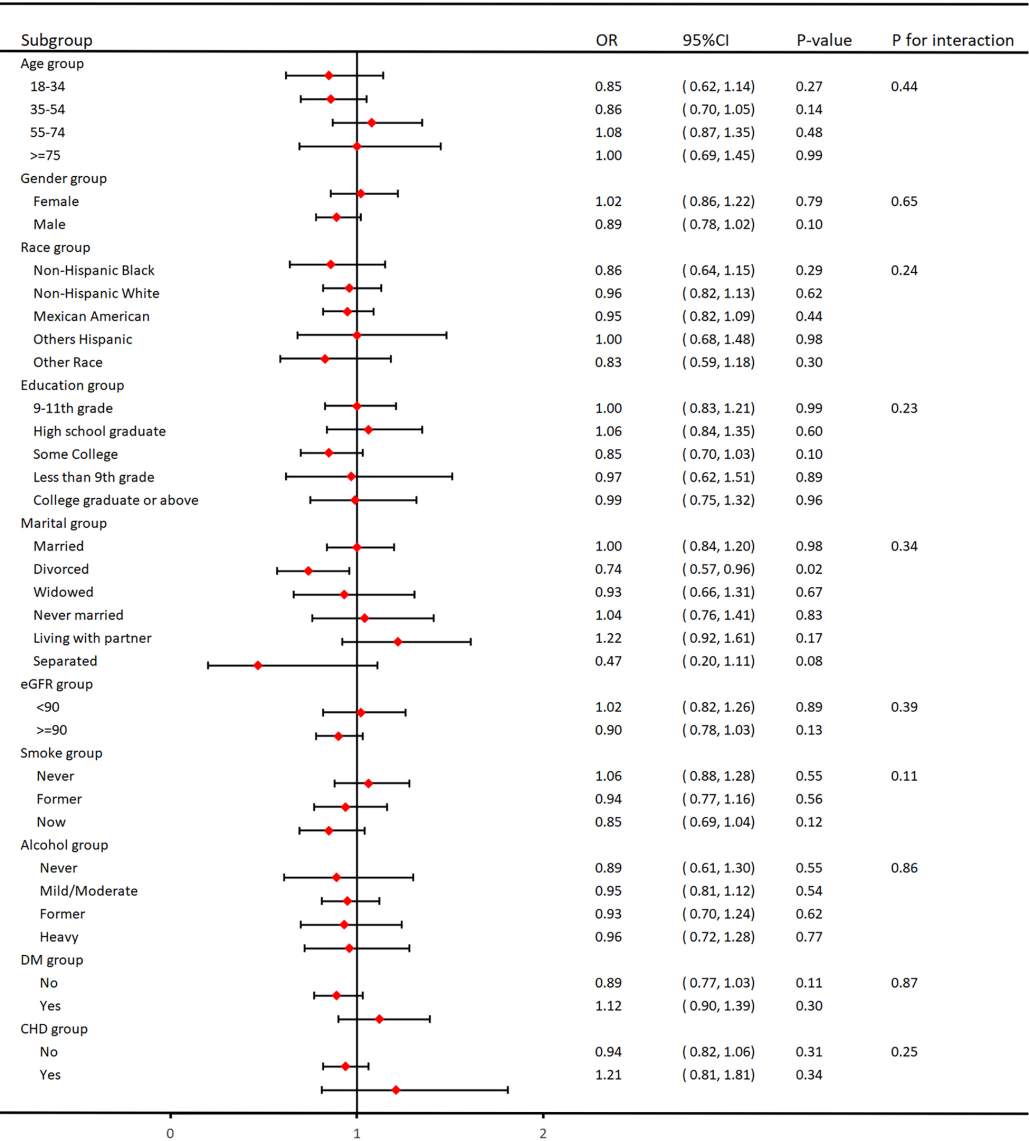
Subgroup analysis for the association between VAI and hypertension.

As shown in Figure 3, we performed subgroup analyses to validate the relationship between VAI and hypertension. It is worth noting that there was no interaction between age, gender, ethnicity, education, marital status, tobacco use, alcohol use, eGFR, DM, and CHD in the subgroups, and they were highly stable.

## 4 Discussion

In this large cross-sectional study, we included 14,569 participants aged ≥18 in the United States to evaluate the link between VAI and hypertension. The outcome shows that in Table 1, the associations between VAI and hypertension were not affected by confounders such as age, race, education, marital relationship, and PIR. Upon adjustment for potential covariates, we discovered a positive relation between VAI and hypertension, suggesting that VAI is a risk factor for the development of hypertension. The greater the level of VAI, The greater the risk of developing hypertension. We performed smoothed curve fitting and found that this positive correlation was nonlinear (*P* for nonlinear <0.001), with a steeper elevation trend with higher VAI values. In addition, subgroup analyses were performed to reveal potential relationships through these data. The subgroups were non-interacting and stable.

The prevalence of overweight and obesity in the population is continuing to rise in the United States and globally, inevitably creating a major epidemic (23, 24). According to epidemiologic statistics, approximately 2.1 billion people worldwide will suffer from the disease by 2030 (25). Obesity is also a significant risk factor for cardiovascular disease, coronary heart disease, type 2 diabetes, heart failure, and hypertension. Although hypertension has been studied in detail, the pathogenesis of obesity is currently unknown. However, it is related to adipose tissue. Adipose tissue dysfunction activates the renin-angiotensin-aldosterone system, which increases blood pressure. First, subcutaneous adipose tissue in the abdomen of obese individuals secretes large amounts of angiotensin II (AngII) (26); second, angiotensinogen overexpression in adipose tissue increases blood pressure (27); and third, adipocytes depend on AngII for aldosterone production (28). Fourth, adipose tissue-derived mineralized corticotropin-releasing factor stimulates aldosterone release in adrenocortical cells (29). Hypertension has also been found to be associated with a variety of hormones secreted in adipose tissue (30). Leptin is an adipocyte-derived hormone, and obesity leads to elevated leptin levels, which stimulates increased sympathetic nerve activity (31), leading to increased heart rate and blood pressure. Resistin is significantly increased during obesity (32), plays a role in insulin resistance (33), and induces hypertension by inducing angiotensinogen (34). Lipocalin secretion is reduced in obese patients (35), leading to a decrease in endothelial nitric oxide synthase and prostaglandin I 2 synthase, which allows for diminished vasodilatation and exhibits elevated blood pressure (36). Therefore, excess adipose tissue may cause hormonal, inflammatory, and endothelial alterations resulting in events such as increased insulin resistance, increased sympathetic activity, activation of the renin-angiotensin-aldosterone system, endothelial dysfunction, and renal sodium reabsorption, which ultimately lead to elevated blood pressure (37).

VAI is a reliable and comprehensive index for assessing visceral fat (11). Visceral fat is measured using abdominal CT or magnetic resonance imaging (MRI). Although these methods are more accurate, they are costly and inefficient, with fewer clinical applications and side effects (17).VAI is calculated from the anthropometric indexes BMI and WC and the biochemical indexes TG and HDL, which are simple to operate and have easily accessible and safe data.VAI is associated with abdominal aortic calcification, cardiovascular disease, metabolic syndrome, and stroke and has been studied epidemiologically (38–41). The present research indicated a nonlinear positive correlation between VAI and the prevalence of hypertension, which may be related to inflammation, insulin resistance and adipocytokine production. Inflammation plays a crucial role in hypertension, and many studies have shown that hypertensive patients have elevated levels of systemic inflammation, and inhibition of inflammation reduces hypertension, which helps to predict the risk of hypertension through inflammation (42). When obesity after excessive calorie intake increases the association between adipose tissue and inflammation, inflammatory expression and systemic inflammation levels increase, allowing vasoconstriction, increased endothelial adhesion, sympathetic excitation and the development of hypertension (43, 44). Visceral adipocytes produce adipocytokines, including leptin, resistin, lipocalin, and inflammatory cytokines, which increase IR (15). Excess adipose tissue can increase IR by promoting inflammation through increased levels of resistin or tumour necrosis factor-α. Adipose tissue macrophages activate inflammatory signalling pathways within neighbouring insulin-targeting cells (adipocytes), releasing inflammatory factors directly involved in the development of IR (45).IR increases the sympathetic nervous system by enhancing angiotensin II (AngII) and aldosterone activity, and oxidative stress leads to elevated blood pressure (46). Therefore, it is advisable to eat a light diet, reduce the intake of high-fat diets, control body weight and blood glucose, enhance exercise, and regularly monitor fat content, blood glucose, and lipid levels to prevent hypertension.

This study has a number of strengths: (i) these are the first cross-sectional investigations to evaluate the link between VAI and hypertension in a widespread American population. (ii) The sample selection and sample size are representative and adequate, and are worthy of replication. (iii) We reviewed related studies and adjusted for the interference of confounders to make the results more accurate. In addition, this study has some limitations: (i) due to the long period, the complicated calculation of VAI, and the fact that the assessment may be subject to anthropometric indicator errors and biochemical indicator inaccuracies. (ii) We could not analyze special populations Because the observation was U.S. adults, excluding minors. (iii) Although confounding factors affecting hypertension were adjusted for in the study, other influencing factors may exist.

## 5 Conclusion

In summary, after adjusting for potential confounders, this cross-sectional study based on eight cycles of NHANE (2003–2018) data showed a nonlinear positive association between VAI and hypertension in US adults. Thus, VAI may serve as a specific marker to identify hypertension risk at an early period in the adult population in order to reduce its prevalence. In the future, more randomized controlled trials or prospective studies are needed to confirm this finding.Secondly, VAI will be extended to basic experiments to explore its pathogenesis with related diseases. Finally, we will promote VAI in the clinic to provide convenience for patients.

## Data Availability

The raw data supporting the conclusions of this article will be made available by the authors, without undue reservation.
